# Stimulatory Effects of Peroxisome Proliferator-Activated Receptor-γ on Fcγ Receptor-Mediated Phagocytosis by Alveolar Macrophages

**DOI:** 10.1155/2007/52546

**Published:** 2007-10-02

**Authors:** David M. Aronoff, Carlos H. Serezani, Jennifer K. Carstens, Teresa Marshall, Srinivasa R. Gangireddy, Marc Peters-Golden, Raju C. Reddy

**Affiliations:** ^1^Division of Infectious Diseases, Department of Internal Medicine, University of Michigan Health System, Ann Arbor 48109, MI, USA; ^2^Division of Pulmonary and Critical Care Medicine, Department of Internal Medicine, University of Michigan Health System, Ann Arbor 48109, MI, USA

## Abstract

Alveolar macrophages abundantly express PPAR-γ, with both natural and synthetic agonists maintaining the cell in a quiescent state hyporesponsive to antigen stimulation. Conversely, agonists upregulate expression and function of the cell-surface receptor CD36, which mediates phagocytosis of lipids, apoptotic neutrophils, and other unopsonized materials. These effects led us to investigate the actions of PPAR-γ agonists on the Fcγ receptor, which mediates phagocytosis of particles opsonized by binding of immunoglobulin G antibodies. We found that troglitazone, rosiglitazone, and 15-deoxy-$\Delta^{12,14}$-prostaglandin ${\text{J}}_{2}$ increase the ability of alveolar, but not peritoneal, macrophages to carry out phagocytosis mediated by the Fcγ receptor. Receptor expression was not altered but activation of the downstream signaling proteins Syk, ERK-1, and ERK-2 was observed. Although it was previously known that PPAR-γ ligands stimulate phagocytosis of unopsonized materials, this is the first demonstration that they stimulate phagocytosis of opsonized materials as well.

## 1. INTRODUCTION

Phagocytosis—engulfment of invading pathogens, particulates, and dying cells—is a crucial homeostatic mechanism in multicellular organisms. Most mammalian phagocytosis is carried out by macrophages or neutrophils. This process begins with adhesion of the material to be phagocytosed to a receptor on the macrophage or neutrophil surface. The receptor then triggers intracellular signals that lead to a zipper-like infolding of the cell membrane, engulfing the receptor and that which is bound to it. Further signals cause transport of the resulting endosome to the lysosome, where enzymes are available to digest commonly phagocytosed materials.

Both oposonin-dependent and-independent classes of cell-surface receptors mediate phagocytosis. Among the former are the Fc receptors that recognize the Fc portion of an immunoglobulin bound through its antigen-recognition site to the target particle or organism [1]. The most important of these is the Fcγ receptor for immunoglobulin G (IgG), but Fcα receptors and Fcε receptors (for the Fc portions of immunoglobulin A and immunoglobulin E, resp.) also exist. Complement receptors also recognize opsonized particles that are bound with complement proteins [2]. The broad class of opsonin-independent receptors involved in immune surveillance and phagocytosis includes the Toll-like and scavenger receptors that recognize apoptotic cells, microbial components, and other unopsonized materials [3, 4].

The nuclear receptor, peroxisome proliferator-activated receptor-γ (PPAR-γ), is expressed in a variety of cells of the immune system, including macrophages, neutrophils, eosinophils, lymphocytes, and mast cells. This receptor is expressed abundantly in alveolar macrophages (AMs) [5–7] but at much lower levels in resident macrophages of the bone marrow and peritoneum [6, 7]. In peritoneal macrophages (PMs) that have been elicited by activating agents such as thioglycolate, however, PPAR-γ is upregulated significantly [7].

Many aspects of AM function have been found to be modulated by both natural and synthetic PPAR-γ ligands [8]. For example, PPAR-γ ligands inhibit the ability of various stimuli to induce production of proinflammatory mediators, including tumor necrosis factor-α and interleukin-12, expression of inducible nitric oxide synthase, and the production of reactive oxygen species [5, 6]. Conversely, activation of PPAR-γ in AMs has been shown to upregulate phagocytosis of apoptotic neutrophils through increased expression of the CD36 surface receptor [5]. PPAR-γ ligands have also been shown to increase CD36-mediated phagocytosis of senescent neutrophils and fluorescent-labeled latex beads by pancreatic stellate cells [9].

In light of these results, we hypothesized that activation of PPAR-γ could regulate Fcγ receptor-mediated phagocytosis. We therefore performed experiments in both AMs and PMs using IgG-opsonized phagocytic targets and ligands for PPAR-γ.

## 2. MATERIALS AND METHODS

### 2.1. Animals

Pathogen-free 129/SvEv mice (The Jackson Laboratory, Bar Harbor, Me, USA) and 125–150 gm female Wistar rats (Charles River Laboratories, Portage, Mish, USA) were utilized. Animals were treated according to National Institutes of Health guidelines for the use of experimental animals with the approval of the University of Michigan Committee on Use and Care of Animals.

### 2.2. Reagents

O-phenylenediamine dihydrochloride, 3-(4,5-dimethyl-thiazol-2-yl)-2, 5-diphenyltetrazolium bromide (MTT), and sodium dodecyl sulfate were obtained from Sigma-Aldrich (St. Louis, Mo, USA). Uniform, superparamagnetic, 2.8 micron polystyrene beads covalently coated with IgG were purchased from Dynal-Invitrogen (Carlsbad, Calif, USA). Troglitazone, rosiglitazone, and 15-deoxy-Δ ^12,14^-prostaglandin J_2_(15d-PGJ_2_) were obtained from Cayman Chemical (Ann Arbor, Mich, USA). These compounds were dissolved in DMSO to a stock concentration of 10 mM and stored at −80^°^C prior to use. RPMI-1640 and penicillin/streptomycin/amphotericin B solutions were purchased from Gibco-Invitrogen (Carlsbad, Calif, USA). Tryptic soy broth was supplied by Difco (Detroit, Mich, USA). *Klebsiella pneumoniae* 43816, serotype 2, was obtained from the American Type Culture Collection (Rockville, Md, USA); aliquots were grown until mid-log phase in TSB at 37^°^C under 5% CO_2_ atmosphere. The concentration of bacteria in culture was determined spectrophotometrically at 600 nm [10]. Required dilutions of all compounds were prepared immediately before use and equivalent quantities of vehicle were added to the appropriate controls.

### 2.3. Cell isolation and culture

Resident AMs from mice and rats were obtained via ex vivo lung lavage as previously described [11] and resuspended in RPMI to a final concentration of $2\times 10^{6}$ cells/mL. Resident peritoneal macrophages (PMs) from mice and rats were harvested by lavage as previously published [12]. Cells were allowed to adhere to tissue-culture-treated slides/plates for 1 hour at 37^°^C in a 5% CO_2_ atmosphere, followed by two washings with warm RPMI to remove nonadherent cells. Prior to use, macrophages were cultured overnight in RPMI containing 10% fetal bovine serum and 1% penicillin/streptomycin/amphotericin B. On the following day, cells were washed again with a warm medium to remove nonadherent cells.

### 2.4. Microcolorimetric erythrocyte phagocytosis assay

Macrophage phagocytosis of IgG-opsonized sheep red blood cells (SRBCs) was assessed as previously described [13, 14]. Briefly, cells were plated and cultured overnight in 96-well culture-treated dishes (Becton, Dickinson, Franklin Lakes, NJ, USA) at a density of $2\times 10^{5}$ cells/well and in the presence of PPAR-γ ligands or vehicle controls. SRBCs (ICN, Costa Mesa, Calif, USA) were opsonized with a subagglutinating concentration of polyclonal rabbit anti-SRBC IgG (Organon Teknika-Cappel, Durham, NC, USA). Macrophages were then washed twice with warm RPMI and preincubated for 45 minutes with cytochalasin D (5 μg/mL) or vehicle. Following preincubation, opsonized SRBCs were added at a ratio of 50 : 1 (SRBC : macrophage) and cultures were incubated for an additional 90 minutes at 37^°^C. Wells were then washed three times with phosphate buffered saline to remove noningested erythrocytes and 100 μL of 0.3% sodium dodecyl sulfate in phosphate buffered saline was added to each well for 10 minutes. A standard curve was derived by adding serial dilutions of known numbers of SRBCs to separate wells followed by addition of sodium dodecyl sulfate solution. Lastly, 100 μL of O-phenylenediamine dihydrochloride solution was added to each well as a chromogen. Following a 30-minute incubation in the dark at 22^°^C, the absorbance (A) at 450 nm was evaluated with an automated reader (VersaMAX, Molecular Devices, Sunnyvale, Calif, USA). The number of SRBCs per well was derived from A_450_ data using the standard curve prepared as described. The phagocytic index (PI) was defined as the number of SRBCs in an experimental well (ingested + adhered SRBCs) minus the mean number of SRBCs in wells treated with the phagocytosis inhibitor cytochalasin D (adhered SRBCs) and was expressed as the percentage of the control. Independent experiments were performed in septuplet.

### 2.5. Phagocytosis of IgG-opsonized beads

Phagocytosis of IgG-opsonized beads (IgG-beads) was quantified via light microscopy. Macrophages were cultured on 8-chamber glass slides before the challenge with IgG-beads at a ratio of 40 beads/cell. PPAR-γ ligands or vehicle controls were added before the addition of IgG-beads as described in Section 3 and/or figure legends. Experiments were terminated and uningested IgG-beads were removed by aspirating supernatants and washing slides three times with cold phosphate buffered saline. Slides were subsequently stained with a modified Wright-Giemsa stain and examined under light microscopy. The PI was determined from 200 cells per well by multiplying the percentage of macrophages containing at least 1 IgG-bead by the mean number of IgG-beads per positive cell [13, 15]. The ability to distinguish intracellular from surface-associated IgG-beads was verified by comparing the PI of untreated cells with that of cells exposed for 30 minutes to the phagocytosis inhibitor cytochalasin D (5 μg/mL) [16]. A minimum of 4 replicate wells per condition was studied in each experiment.

### 2.6. Phagocytosis of live, serum-opsonized bacteria

Once the Gram-negative pathogen *K. pneumoniae* has been opsonized with immune serum, it is subject to phagocytosis by alveolar macrophages via the Fcγ class of receptors [17]. We assessed phagocytosis of *K. pneumoniae* based on a protocol for bacterial killing that we have previously published [18]. Briefly, rat AMs at a concentration of $2\times 10^{6}$/mL, prepared as described, were seeded in a 96-well tissue culture dish and exposed to PPAR-γ ligands or vehicle controls for 18 hours. The next day, *K. pneumoniae* were opsonized with 3% anti-*K. pneumoniae* rat-derived immune serum, as previously described [16]. Macrophages were then infected with a 0.1-mL suspension of opsonized *K. pneumoniae* ($1\times 10^{7}$ colony-forming units/mL; multiplicity of infection, 50 : 1) and incubated for 30 minutes to allow phagocytosis to occur. Cells were then washed three times with 100 μL of phosphate buffered saline to remove noningested bacteria, after which the macrophages were lyzed with 100 μL of TSB containing 0.5% saponin (which did not lyze the bacteria). Cultures were incubated for 2 hours at 37^°^C to amplify bacterial growth prior to the addition of the tetrazolium salt MTT (5 mg/mL in phosphate buffered saline). Plates were held for 30 minutes at 37^°^C, after which the purple formazan salt was solubilized with a solution of isopropanol/0.1 N HCL and 1% Triton X-100 [19]. The intensity of the absorbance at 595 nm was directly proportional to the number of intracellular bacteria associated with the macrophages [19]. Results are expressed as a percent of the untreated cells.

### 2.7. Immunoblot analysis

Western blots were performed as previously described [20]. Briefly, the whole cell protein extracts were obtained by lyzing freshly harvested AMs in a buffer [50 mM Tris-HCl (pH 7.4), 25 mM KCl, 5 mM MgCl_2_, 0.2% Nonidet P-40] supplemented with protease and phosphatase inhibitors (Roche Diagnostics, Mannheim, Germany). Protein samples (40 μg) were resolved on 10% Tris-HCl polyacrylamide gels and subsequently transferred to nitrocellulose membranes. Membranes were probed with commercially available rabbit polyclonal antibodies against phospho-spleen tyrosine kinase (phospho-Syk; Tyr525/526; Cell Signaling Technology, Danvers, Mass, USA; 1 : 500), total Syk (Santa Cruz Biotechnology, Inc., Calif, USA; 1 : 800), total p42/44 (ERK-1/2; Cell Signaling Technology; 1 : 1000), or with mouse monoclonal antibodies against β-actin (Sigma-Aldrich; 1 : 10000) or phospho-p42/44 (Tyr204/Thr202; Cell Signaling Technology; 1 : 1000) followed in either case by horseradish peroxidase-conjugated antirabbit or antimouse, respectively, secondary antibodies, and ECL chemiluminescence detection reagents (Amersham Biosciences, Piscataway, NJ, USA). For experiments involving activation of the Fcγ receptor, AMs were treated for 7 minutes with IgG-SRBCs at a ratio of 33 SRBC per macrophage [21]. Band density from Western blots was determined using Adobe Photoshop 6.0 (Adobe, San Jose, Calif, USA).

### 2.8. RT-PCR of Fcγ receptors I, IIB, and III

The mRNA expression of Fcγ receptors I, IIB, and III was determined in macrophages treated for 16 hours with troglitazone (5 *μ*M) or with DMSO vehicle. RNA from cultured cells was isolated using the RNeasy Mini Kit (Qiagen, Hilden, Germany) according to the manufacturer's instructions. RT-PCR was then performed using the Access RT-PCR kit (Promega Corporation, Madison, Wis, USA) according to the manufacturer's directions, with 100 ng of RNA being used for each reaction. The primers used in the reaction were synthesized according to standard methods and displayed in Table 1. The PCR conditions were as follows: 45 minutes at 45^°^C, 2 minutes at 94^°^C followed by 30 cycles of 30 seconds at 94^°^C followed by 1 minute at 58^°^C, and then 90 seconds at 68^°^C. All PCRs were performed in a reaction volume of 50 μL.

### 2.9. Statistical analysis

Data are represented as mean ± SE and were analyzed with the Prism 4.0 statistical program (GraphPad Software, San Diego, Calif, USA). Comparisons between two experimental groups were performed with Student *t* test. Comparisons among ≥3 experimental groups were performed with analysis of variance (ANOVA) followed by Dunnett's adjustment for multiple comparisons. Differences were considered significant if P<.05. All experiments were performed on at least three separate occasions unless otherwise specified.

## 3. RESULTS

### 3.1. Troglitazone increases Fcγ receptor-mediated phagocytosis in rat AMs but not PMs

**Figure 1:**
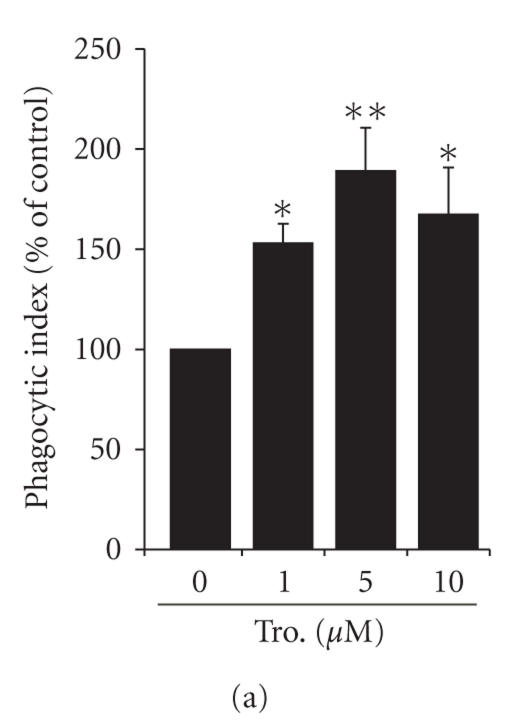

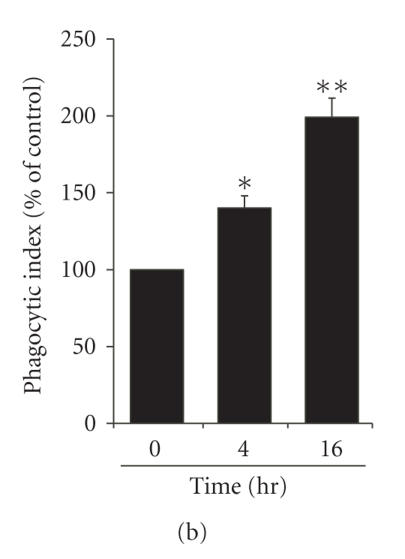

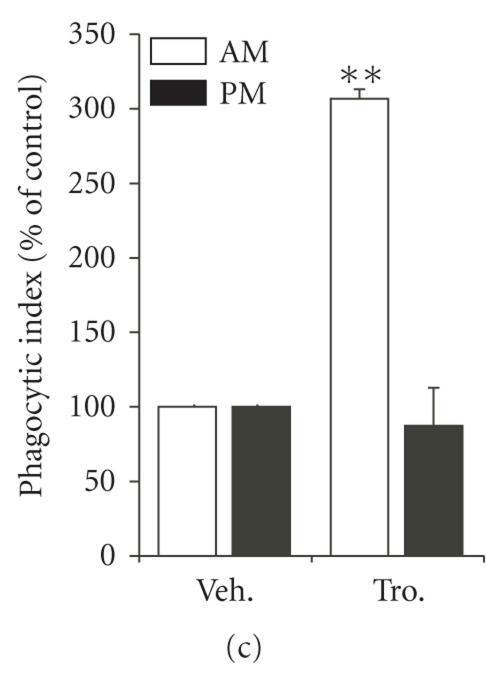
Stimulation of Fcγ receptor-mediated phagocytosis by troglitazone in rat macrophages. Rat alveolar macrophages (AMs) were treated for 16 hours with troglitazone at the doses indicated by (a) or with 5 *μ*M troglitazone for the times indicated by (b) prior to the challenge with IgG-opsonized sheep red blood cells (SRBCs). In (c), both AMs and peritoneal macrophages (PMs) were treated for 16 hours with 5 *μ*M troglitazone before phagocytosis was assessed, as described in Section 2. ^*^ P<.05 and ^**^ P<.01 compared to untreated cells.

Troglitazone is a thiazolidinedione no longer approved for human use but still commonly used experimentally to activate PPAR-γ. An earlier study demonstrated that doses > 10 μ
M decreased Fcγ receptor-mediated phagocytosis in a macrophage-like cell line, although this effect was accompanied by apoptosis [22]. To study Fcγ receptor-mediated phagocytosis in a more biologically relevant system, we employed lower, nonapoptotic inducing doses of this drug using primary AMs. As shown (Figures 1(a) and 1(b)), troglitazone enhanced the ingestion of IgG-SRBCs by rat AMs, with the effect being both dose- and time-dependent. The peak effect occurred with a 16-hour incubation in the presence of 5 μM troglitazone; this exposure increased phagocytosis to 199 ±12.4% of the untreated value (Figure 1(b)). At this dose of troglitazone, apoptosis was not observed (data were not shown).

Unlike AMs, PMs express little PPAR-γ [6]. We speculated that the effect of troglitazone would be more potent in AMs than PMs, reflecting the differences in PPAR-γ expression. Indeed, we observed no increase in the ingestion of IgG-SRBCs by rat PMs treated with troglitazone (Figure 1(c)).

### 3.2. Troglitazone enhances Fcγ receptor-mediated phagocytosis in murine AMs but not PMs

**Figure 2:**
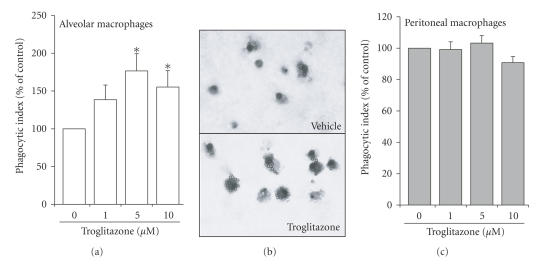
Stimulation of Fcγ receptor-mediated phagocytosis by troglitazone in mouse macrophages. Murine AMs (a) and (b) or PMs (c) were treated for 16 hours with 5 μM troglitazone before phagocytosis of IgG-opsonized beads was assessed, as described in Section 2. Panel (b) is a representative light microscopy field (400x magnification) demonstrating the effect of troglitazone (5 μM, bottom panel) compared to vehicle (top panel). ^*^ P<.05 compared to untreated cells.

To address the generalizability of our initial observation, we repeated our experiments using murine macrophages and a different IgG-opsonized target, an IgG-coated polystyrene bead. Figure 2 demonstrates that troglitazone enhanced Fcγ receptor-mediated phagocytosis by AMs over the same concentration range observed for the rat, while no effects were seen in the PMs.

### 3.3. Multiple PPAR-γ ligands enhance Fcγ receptor-mediated phagocytosis by AMs

**Figure 3:**
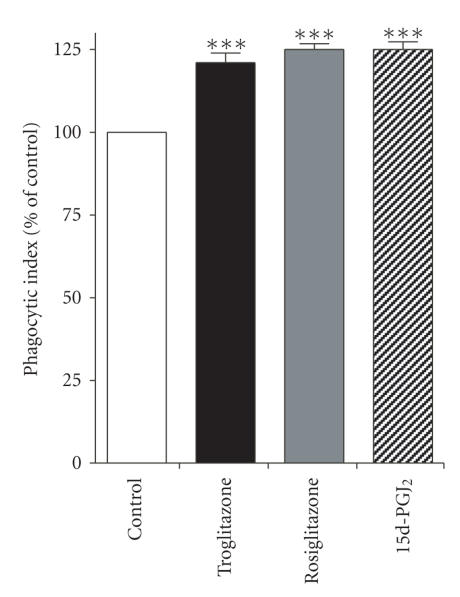
PPAR-γ ligands enhance phagocytosis of opsonized *K. pneumoniae*. Rat AMs were pretreated for 16 hours with troglitazone, rosiglitazone, or 15d-PGJ_2_ (each at 10 μM) prior to infection with immune serum-opsonized *K. pneumoniae* at a multiple of infection of 50 : 1. Phagocytosis was determined after 30 minutes, as detailed in Section 2. ^***^ P<.001 compared to untreated cells.

The above studies were limited by (a) the application of a single PPAR-γ ligand with known/suspected PPAR-γ-independent signaling properties [23] and (b) the use of nonphysiological targets of IgG-opsonization. We therefore tested the ability of rat AMs to ingest IgG-opsonized bacteria using the relevant Gram-negative pathogen *K. pneumoniae*. As demonstrated in Figure 3, troglitazone, rosiglitazone, and 15d-PGJ_2_ each increased phagocytosis of *K. pneumoniae* by ∼20%–25% when administered to the cells at a 10 μM concentration. Thus, distinct PPAR-γ ligands enhance the ingestion of IgG-opsonized pathogens by primary lung macrophages.

### 3.4. PPAR-γ activation does not modulate Fcγ receptor expression

**Figure 4:**
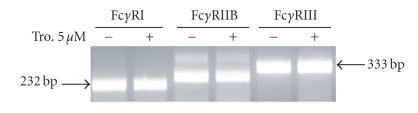
Expression of mRNA for Fcγ receptors is not affected by PPAR-γ ligands. Mouse AMs were plated and treated for 16 hours with either 5 μM troglitazone or DMSO vehicle. RNA was isolated, amplified by RT-PCR, and subjected to electrophoresis. The expected sizes of cDNAs for Fcγ receptors I, IIB, and III, respectively, are 232, 277, and 333 bp.

PPAR-γ ligands have been shown to increase the phagocytosis of apoptotic cells by increasing the cell surface expression of the CD36 receptor [5]. By analogy, we speculated that the observed stimulation of Fcγ receptor-mediated phagocytosis by PPAR-γ ligands might reflect increased expression of that receptor. We therefore performed RT-PCR for the Fcγ receptors I and III using RNA extracted from mouse AMs treated for 16 hours with 5 μM troglitazone. We also considered an alternative possibility that PPAR-γ activation might suppress the expression of the Fcγ IIB receptor, which is an inhibitory Fcγ receptor. However, we did not detect significant differences in the expression of any of these three receptors by RT-PCR (Figure 4), confirming the flow-cytometric results obtained by Kasono et al. using J774.A1 macrophages [22].

### 3.5. Troglitazone enhances post-Fcγ receptor signaling in AMs

**Figure 5:**
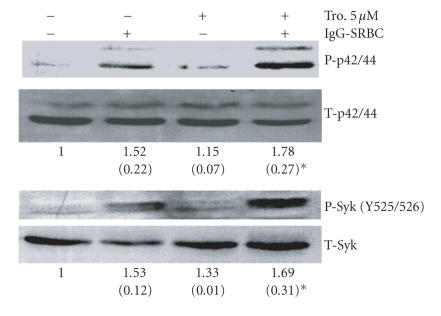
Troglitazone enhances Syk and ERK activation during Fcγ receptor-mediated phagocytosis. Rat AMs were treated with 5 μM troglitazone for 16 hours prior to challenge with IgG-opsonized SRBCs. Unopsonized SRBCs were used as negative controls. Cells were lyzed after 7 minutes and subjected to Western immunoblot analysis. Bands labeled p42 and p44 represent ERK-1 and -2. The phosphorylation of Syk was identified on the tyrosine residues 525 and 526. Representative blots from three independent experiments are shown. Values represent the mean (± SE) of the ratio of phosphyorylated to total proteins determined by band densitometry from multiple experiments (n=3−4), expressed relative to untreated cells. ^*^ P<.05 compared to untreated cells.

Because the expression of Fcγ receptors was not altered by troglitazone, we postulated that PPAR-γ activation might be enhancing the intracellular signaling network involved in the internalization of IgG-opsonized targets. We therefore tested the effect of troglitazone (5 μM for 16 hours) on the activation of proximal and distal signaling molecules involved in Fcγ receptor-mediated phagocytosis [24]. As shown in Figure 5, the proximal tyrosine kinase Syk becomes phosphorylated when cells are challenged with IgG-SRBCs; this phosphorylation was significantly enhanced by troglitazone. The extracellular signal-regulated protein kinases (ERK)-1 and -2 (also known as p42/44 proteins) are also important in IgG-mediated phagocytosis [24]. We found that 16-hour administration of troglitazone to AMs stimulated activation of ERK-1 and -2 over and above that triggered by IgG-SRBCs alone. Analysis showed that only prior treatment with troglitazone led to statistically significant increases in the phosphorylation of Syk or ERK proteins in response to opsonized SRBCs (Figure 5).

## 4. DISCUSSION

In this study, we demonstrate that activation of PPAR-γ enhances the phagocytosis of IgG-opsonized targets via the Fcγ class of receptors in AMs. To our knowledge, this is the first study to demonstrate that PPAR-γ ligands increase Fcγ receptor-mediated phagocytosis. Notably, the effects of troglitazone that were seen in AMs were not observed in resident PMs. This result accords with the earlier finding that AMs express significantly more PPAR-γ than PMs do [6].

We hypothesized that PPAR-γ activation might regulate Fcγ receptor-mediated phagocytosis based on the nuclear receptor's known ability to enhance phagocytosis mediated via other receptors on the cell surface. For example, PPAR-γ activation has been shown to increase expression of the cell surface receptor CD36, which is involved in the recognition and internalization of apoptotic cells, and thereby to enhance apoptotic cell uptake by macrophages [5]. The phagocytosis of senescent neutrophils and unopsonized polystyrene beads by pancreatic stellate cells was also enhanced by PPAR-γ-activating agents [9]. This effect was also shown to result from increased expression of the cell-surface receptor CD36, although the receptor(s) involved was not specifically characterized.

Our studies were strengthened by the use of AMs and PMs from both rats and mice and by the use of multiple IgG-opsonized targets, including standard SRBCs and live bacterial pathogens. However, our results appear to differ from the only other published study of PPAR-γ activation and receptor-mediated phagocytosis [22]. Using the macrophage-like cell line J774.A1, Kasono et al. found that troglitazone, pioglitazone, and 15d-PGJ_2_ suppressed phagocytosis of IgG-opsonized SRBCs without—as we also found—altering Fcγ receptor expression. However, the authors demonstrated that both troglitazone and pioglitazone induced significant apoptosis in these cells at the same concentrations used to suppress phagocytosis (15d-PGJ_2_ was not tested). It therefore seems likely that the inhibition by PPAR-γ ligands of Fcγ receptor-mediated ingestion in J774.A1 cells occurred primarily as a consequence of cell death through apoptosis. It is notable, however, that Kusano et al. found that both the suppression of phagocytosis and the induction of apoptosis occurred at doses of troglitazone > 30 μM, whereas a dose of 10 μM caused an increase in phagocytosis that did not reach statistical significance. We also observed inhibition of phagocytosis and cell death in AMs at concentrations of troglitazone > 10 μM (data were not shown).

Although we found qualitatively similar, stimulatory effects of troglitazone on Fcγ receptor-mediated phagocytosis using three unique phagocytic targets (erythrocytes, beads, and *K. pneumoniae*), the magnitude of troglitazone's effects differed with regards to the model examined. The reasons for this are not entirely clear. The greatest effect of troglitazone was seen in assays using inert targets (IgG-SRBCs and IgG-beads), as compared to the use of live, serum-opsonized bacteria. We speculate that as yet undefined differences between the interactions of macrophages with live bacteria versus interactions with inert targets might underlie these variabilities.

Azuma et al. demonstrated that the PPAR-γ ligand 15d-PGJ_2_ dose dependently inhibited the phagocytosis by glycogen-elicited (activated) PMs from Wistar rats of unopsonized *Escherichia coli* [25] (lack of opsonization implied that phagocytosis was not mediated by the Fcγ receptor). However, since PPAR-γ expression is known to be markedly upregulated in activated compared to resident PMs [7], the disparity between these results and our failure to find an effect of troglitazone on phagocytosis via the Fcγ receptor in resident PMs is not surprising.

The finding of activation of phagocytosis in AMs, rather than the inhibition that Azuma et al. observed in activated PMs, may be attributed to differences in the two cell types [26, 27]. The alveolus is constantly exposed to pathogens and irritant particles drawn in with the inspired air, and the inciting of an inflammatory response to inhaled irritants might impair the ability of the alveolar space to participate in the essential function of gas exchange. Studies have shown that PPAR-γ ligands inhibit AM inflammatory responses, including the production of reactive oxygen species, and the expression of pro-inflammatory cytokines and inducible nitric oxide synthase [5, 6]. Phagocytosis without accompanying inflammatory activity, however, does not threaten alveolar function. There is, thus, no conflict between downregulation of inflammatory responses and simultaneous upregulation of phagocytosis mediated by either CD36 receptors [5, 9] or Fcγ receptors (this study). This point is further supported by the finding of Sutterwala et al. in bone marrow macrophages, in which the binding of materials such as IgG-opsonized SRBCs to the Fcγ receptor promoted the production of the anti-inflammatory cytokine interleukin-10 and the resultant inhibition of the pro-inflammatory cytokine interleukin-12's production [28].

Regulation of Fcγ receptor expression and activity is complex. Granulocyte-macrophage colony stimulating factor is required both for constitutive expression in AMs and for upregulation of receptor expression by interferon-γ [29]. Mancuso et al. found that leukotrienes B_4_ and C_4_, as well as 5-hydroxyeicosatrienoic acid (5-HETE), stimulated AM phagocytosis of *K. pneumoniae* [16]. A subsequent study showed that this effect was specific to bacteria opsonized with IgG and due to downstream activation of Fcγ receptor internalization and transport rather than to increased receptor expression [17]. These same leukotrienes stimulate AM bactericidal activity by activating NADPH oxidase and stimulating production of H_2_O_2_ [18], an effect that in this case is opposite to that of PPAR-γ ligands. Conversely, reflecting the frequent antagonism between leukotrienes and prostaglandins, prostaglandin E_2_ has been shown to inhibit Fcγ receptor-mediated phagocytosis in AMs [13].

We found that, just as with leukotrienes, increased Fcγ receptor-mediated phagocytosis induced by PPAR-γ ligands did not result from increased receptor expression. While the PPAR-γ ligands did not alter Fcγ receptor mRNA expression, these studies do not rule out the possibility that the ligands altered phagocytosis by increasing the surface expression of these receptors. Regardless, we infer from our data that PPAR-γ ligands prime cells for an enhanced activation of downstream effectors involved with postbinding internalization and transport, such as Syk, ERK-1, and ERK-2. While our data support a mechanism whereby PPAR-γ ligands stimulate post-Fcγ receptor signaling (rather than receptor expression), our work does not definitively establish the true role of these signaling pathways in this process.

Syk, which is a protein tyrosine kinase, has been shown to be essential for the transport of internalized Fc receptors to lysosomes [30]. Enhancement of Fcγ receptor-mediated phagocytosis in AMs by LTB_4_ has also been shown to depend on Syk activation [21]. Our study appears to be the first to demonstrate effects of PPAR-γ ligands on Syk activity. Effects of PPAR-γ ligands on ERK-1/2 activation, however, have previously been established. For example, inhibition of growth and induction of apoptosis by 15d-PGJ_2_ in a neuroblastoma cell line was associated with ERK activation [31] as was troglitazone-induced arrest of cell growth in lung adenocarcinoma cells [32]. Similar effects of troglitazone in lung cancer cells were shown to be blocked by inhibition of either PPAR-γ expression or ERK-1/2 activity [33]. In a mouse osteoblastic cell line, induction of apoptosis by ciglitazone was accompanied by increased amounts of phosphorylated ERK, with cell death being blocked by both PPAR-γ and ERK antagonists [34].

It may be questioned whether the effects we saw were necessarily mediated via PPAR-γ, since it is known that 15d-PGJ_2_ and thiazolidinediones can act through PPAR-γ-independent mechanisms [35, 36]. Although the evidence is indirect, finding similar effects with troglitazone, rosiglitazone, and 15d-PGJ_2_ argues for an effect mediated through their common receptor. This conclusion is further strengthened by the observation that such effects were seen in AMs, where PPAR-γ expression is abundant, but not in resident PMs that express relatively little of this receptor.

In summary, we demonstrate here that PPAR-γ ligands stimulate phagocytosis via the Fcγ receptor in AMs but not in PMs. This effect does not depend on increased expression of the cell-surface receptor, but rather on downstream activation of Syk, ERK-1, and ERK-2. In AMs, PPAR-γ ligands thus stimulate phagocytosis mediated by two quite different classes of cell-surface receptors and do so via quite different mechanisms.

## Figures and Tables

**Table 1:** Primer sequences used for RT-PCR.

Gene	Primer	
FcγRI	Forward	5^′^-GAG CAG GGA AAG AAA GCA AAT TCC-3^′^
	Reverse	5^′^-TTA AGA GTT GCA TGC CAT GGT CC-3^′^ (232 bp)
FcγRIIB	Forward	5^′^-CCC AAG TCC AGC AGG TCT TTA CC-3^′^
	Reverse	5^′^-TTC TGG CTT GCT TTT CCC AAT GCC-3^′^ (277 bp)
FcγRIII	Forward	5^′^-GAT CCA GCA ACT ACA TCC TCC ATC-3^′^
	Reverse	5^′^-GCC TTG AAC TGG TGA TCC TAA GTC-3^′^ (333 bp)
